# An Open-source Static Threshold Perimetry Test Using Remote Eye-tracking (Eyecatcher): Description, Validation, and Preliminary Normative Data

**DOI:** 10.1167/tvst.9.8.18

**Published:** 2020-07-13

**Authors:** Pete R. Jones

**Affiliations:** 1 University College London (UCL), Institute of Ophthalmology, London, UK; 2 NIHR Moorfields Biomedical Research Centre, London, London, UK; 3 City, University of London, School of Health Sciences, Division of Optometry and Visual Sciences, London, UK

**Keywords:** visual field, standard automated perimetry, static perimetry, eye-tracking, tangent screen, threshold, luminance sensitivity, hill of vision, ZEST, field loss

## Abstract

**Purpose:**

To describe, validate, and provide preliminary normative data for an open-source eye-movement perimeter (Eyecatcher).

**Methods:**

Visual field testing was performed monocularly in 64 normally sighted young adults, using (i) a Humphrey Field Analyzer (HFA) and (ii) the novel Eyecatcher procedure. Eyecatcher used a remote eye-tracker to position stimuli relative to the current point of fixation, and observers responded by looking towards the stimulus. In both tests, Goldman III stimuli were sampled from a 24-2 grid, and were presented against a 10 cd/m^2^ background. Participants completed each test twice to assess test–retest repeatability.

**Results:**

Mean Sensitivity (MS) did not differ between Eyecatcher and the HFA (*P* = 0.086), and both tests exhibited similar test–retest repeatability (CoR_Eyecatcher_ = ±1.86 dB; CoR_HFA_ = ±1.95 dB). Eyecatcher was also able to detect changes in sensitivity across the normal visual field (the “Hill of Vision”), and could differentiate the physiological blind spot from adjacent retinal locations. Mean sensitivities and 95% limits of agreement are described for each pointwise location.

**Conclusions:**

Eyecatcher can use eye movements to assess visual fields in young, normally sighted adults. In such observers, it provides results similar to the current gold standard clinical device (HFA).

**Translational Relevance:**

Given further development, eye movement perimeters such as Eyecatcher could be particularly useful for individuals unable to perform traditional perimetric assessments, such as young children or stroke patients. Full technical details and information on how to freely acquire the source code are included.

## Introduction

Visual field loss affects 3% of 16- to 60-year-olds, and 13% of those over 60 years of age.^1^ It is a key marker for many eye diseases^2^^–^^12^ and correlates with everyday quality of life,^13^^–^^15^ as well as with wider visual function.^16^^–^^20^

Visual field loss is assessed clinically using standard automated perimetry (SAP). With current SAP devices (e.g., Octopus, Dicon, Henson Perimeters, or the Humphrey Field Analyzer [HFA]), the patient's head is fixed in place using a chin rest. Patients are then asked to (i) fixate a central target, and (ii) press a button when they see a white light appear. Lights are presented at one of *n* discrete locations, and an automated algorithm is used to adapt successive light intensities to determine the luminance detection threshold for each location.

Existing SAP procedures require steady fixation, sustained attention, and an explicit button press response. They are therefore inappropriate for young children,^21^^–^^23^ many of whom “are often unable to remain still on a chin rest, unable to suppress their natural looking response to the peripheral target, or [to] give a voluntary, learned response.”^24^ They are also inappropriate for some adult patients, such as those with developmental delay, neurologic impairments, or limited motor function (e.g., following stroke). In these cases, clinicians are limited to qualitative assays such as confrontation testing, with limited sensitivity.^25^^,^^26^

Modern eye-tracking technology offers a potential means to objectively quantify visual field loss in otherwise difficult-to-test populations. By monitoring gaze in real time, stimuli can be presented relative to the patient's current point of fixation. Patients are therefore free to move their eyes during testing and do not have to maintain fixation on a central target. Eye movements toward the stimuli can also be used to determine whether the stimulus was seen: an intuitive response that is present from birth^27^ and which does not require use of arms or hands. Eye-movement perimetry is therefore ideal for use with infants^28^ or patients with impaired motor function, who could not otherwise complete standard (button press) SAP assessments. Finally, modern remote eye trackers are also able to monitor the location of the patient's head. Any changes in viewing distance can therefore be accounted for, by dynamically scaling the size of the stimulus in near real time. This functionality removes the need for chin rests, which many patients find uncomfortable,^29^ and which are inappropriate for infants or the infirm.

In the present article, we describe a novel threshold perimeter (Eyecatcher), that combines inexpensive eye-tracking technology with a rapid adaptive algorithm.^30^^–^^33^ This, and other similar technologies, have been used previously to perform suprathreshold screening.^23^^,^^34^^–^^40^ However, the ability to quantify thresholds is important for the early detection of many blinding diseases^41^ and is vital for evaluating new treatments and monitoring disease progression. Furthermore, unlike previous similar tests,^42^ Eyecatcher is a noncommercial enterprise, and all of the source code associated with it is freely available online (see Supplemental Material). Here we describe the test, validate it, and provide preliminary normative data in a sample of normally sighted adults.

## Methods

### Overview

Sixty-four adults with normal or corrected-to-normal vision completed four perimetric examinations within a single session. Participants were tested twice using a commercial HFA device, and twice using the novel Eyecatcher procedure. All four examinations were interleaved (ABAB) within a single session, with the starting method counterbalanced randomly between participants (16 HFA first, 16 Eyecatcher first). All examinations were carried out monocularly using the same eye, and test eye was counterbalanced randomly between participants (16 left eye, 16 right eye). Testing was carried out under mesopic ambient lighting conditions (HFA: 0.09 lx; Eye-tracking: 0.07 lx), as measured using an Amprobe LM-120 Light Meter (Danaher Corporation, Washington, DC).

### Participants

Participants were 64 young adults (43 female), aged 19.1 to 31.0 years (mean, 24.3 years), with no previous experience of visual field testing. No formal sample size calculation was performed in advance. However, a post hoc analysis indicated that this sample size was sufficient to detect a 0.55 dB or greater difference in MS between Eyecatcher and the HFA, given a two-tailed α (type 1 error) rate of 0.05 (and given the observed standard deviation of paired-differences of 1.548 dB).^43^

Participants wore their own habitual refractive correction (glasses or contact lens) as required. Normal vision was assessed by letter chart acuity (all ≤0.2 logMAR: logarithm of the minimum angle of resolution) and self-report medical histories. An additional six people (9%) were recruited, but could not complete the test because their eyes could not be tracked reliably. Likewise, during piloting three of 46 individuals (7%) could not be tracked reliably. These 46 individuals were asked to perform various earlier version of Eyecatcher, and their data are not reported. See the Discussion for further consideration regarding the issue of tracking failures. Three of the 64 participants in the main experiment also completed a number of post-hoc validation tests, as detailed in the Supplemental Material.

All participants were recruited through the UCL Psychology Department participant pool, and received £7 compensation for their time. The research was carried out in accordance with the Declaration of Helsinki, and was approved by the UCL Ethics Committee (#8650/001). Informed, written consent was obtained from all participants before testing.

### HFA Hardware and Procedure

HFA testing was performed using a HFA II: Model 740i (Carl Zeiss Meditec Inc., Dublin, CA). Participants completed a standard 24-2 Threshold test, using Goldmann III/0.43° stimuli, the SITA standard thresholding algorithm, and a 10 cd/m^2^ white background. For ease of comparison, pointwise sensitivity values (in dB units of attenuation) were converted to the same scale as Eyecatcher, as described elsewhere in this article (see Measurements of Luminance Sensitivity).

### Eyecatcher Hardware

**Figure 1.**
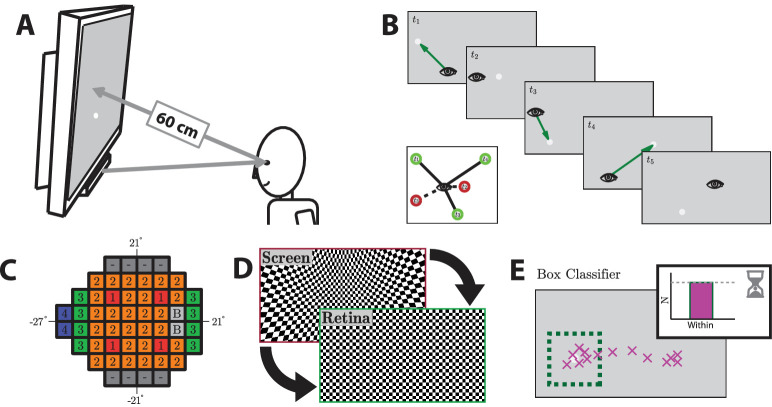
Eyecatcher hardware and procedures. (A) Hardware. Stimuli were presented on a 59.7 × 33.6 cm (2560 × 1440 pixel) LCD screen, which was viewed at a distance of approximately 60 cm (i.e., 52.9° × 31.3° visual angle). A Tobii EyeX eye-tracker was mounted below the screen and was used to measure the gaze and head position relative to the screen. This allowed stimuli to be placed so that they fell at set locations on the retina, and to evaluate eye movement responses. Head position and gaze location were unconstrained, and accounted for in software. (B) Trial sequence. Goldmann III targets of variable intensity were placed relative to the current point of fixation. Note that, owing to the brief presentation time (200 ms, as per perimetric convention), the target was therefore not visible by the time any saccades were complete.^49^ (C) Test grid and growth pattern. Targets were located on a 24-2 perimetric grid. The ZEST algorithm tested groups of points in the order shown, and used both normative data and estimates from earlier test points to inform starting values. The blind spot points (“B”) were tested throughout, independent of the growth pattern. The top and bottom rows were omitted. See the Supplemental Material for details (D) Stimulus warping. A corrective distortion was applied (in software) to ensure a constant stimulus size/shape on the retina, despite the use of tangent-screen presentation. For example, stimuli in the far periphery of the screen were physically larger (in pixels) than those presented centrally. (E) Eye movement classification. Responses were deemed a ‘hit' if *N* gaze estimates (purple crosses; sampled at 50 Hz from the eye-tracker) fell within a *D°* × *D*° box centered on the target location (green dashed line), within *R* seconds of stimulus onset. The parameters *N*, *D*, and *R* varied as a function of stimulus eccentricity (e.g., for a target at 〈+9°, +9°〉: *N* = 6, *D* = 2.77, *R* = 1.62). See the Supplemental Material for further details.

Eyecatcher hardware (Fig. 1A) consisted principally of: an ordinary desktop computer, running Windows 7 (Microsoft Corporation, Redmond, WA); a 10-bit LCD (IPS) monitor (EIZO CG277; EIZO Corporation, Hakusan, Ishikawa, Japan); a 10-bit graphics card (Nvidia Quadro K620; Nvidia Corporation, Santa Clara, CA); and a near-infrared remote eye-tracker (Tobii EyeX; Tobii Technology, Stockholm, Sweden). Note, however, that Eyecatcher procedure is not dependent on any specific equipment, and pilot data were also gathered using various other eye trackers and visual displays (see the Supplemental Material for discussion and recommendations).

Stimuli were generated in Matlab R2014b (32-bit; The MathWorks, Natick, MA) using Psychtoolbox v3.0.11.^44^^,^^45^ Eye-tracking data were retrieved from the Tobii EyeX engine (v1.2.0) using custom *C* code,^46^ and were processed in Matlab using a custom toolbox (‘IVIS’), which is included in Eyecatcher distribution. The code for Eyecatcher is freely available online under a GNU GPL v3.0 license (https://github.com/petejonze/Eyecatcher).

### Eyecatcher Procedure

As with the HFA, targets were 0.43° diameter (Goldmann III) circles of variable luminance, presented on a 24-2 grid, against a 10 cd/m^2^ white background (Fig. 1C). However, unlike the HFA:

1. Participants responded by making an eye movement toward the target location, rather than by pressing a button (see Fig. 1E). They were not given any practice or detailed instructions, and were told simply to “look at anything that appears on the screen.”
2. Participants were not required to maintain fixation on a central cue. Instead, target stimuli were presented relative to the current point of fixation, wherever the participant was fixating at trial onset (Fig. 1B). Typically, this would be the target location from the previous trial.
3. Participants sat normally on a standard office chair and head location was not constrained. The eye tracker measured eyeball location, and so was able to stabilize gaze location, independent of head movements. In addition, trial-by-trial estimates of viewing distance were used to dynamically adjust the size of the stimulus on the screen, to ensure a constant stimulus size on the retina. Participants could therefore shift position, without confounding the results of the test. Note that the ∼60 cm viewing distance is farther than the standard SAP viewing distance of 33 cm. This is primarily due to the eye-tracking hardware, which is set to focus at a distance of approximately 50 to 70 cm.
4. As shown in Figure 1C, the four test points from the top and bottom of the standard 24-2 grid were omitted. This was due to technical limitations; current eye trackers tend to have limited vertical range and often exhibit poor precision and systematic inaccuracies in the vertical extremities.
5. Because the HFA's thresholding algorithm (SITA standard) is proprietary technology, the qualitatively similar ZEST algorithm^30^^–^^33^ was used to adapt stimuli and determine detection thresholds. The prior was a bimodal probability density function, constructed by combining normative data for healthy and glaucomatous eyes, as per Turpin et al.^33^ The likelihood function was a cumulative Gaussian, with a fixed slope of σ = 1.25, and a variable mean of µ = 〈0, 1, 2, …, 34〉. The growth pattern is given in Figure 1C. A dynamic termination criterion was used,^47^^,^^48^ in which the spread of the estimated posterior function was required to have a standard deviation of σ ≤ 1.5 dB.
6. To remove stimulus edge effects (a potential detection artefact), targets were blurred at the edges (Gaussian profile; see the Supplemental Material).

**Figure 2.**
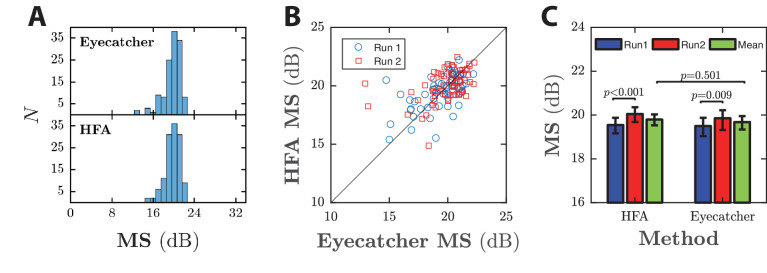
Estimates of mean luminance sensitivity, MS, given the novel eye tracking procedure and the gold standard HFA. (A) Histograms showing the distribution of results for all 128 (64 × 2) tests. (B) Scatter plot showing within-participant correlations. Markers show MS scores for the first (blue circles) and second (red squares) test run. The dashed black line is the identity line: data falling along this line would indicate perfect agreement between the two tests. (C) Group mean data (±95% CI), for the first and second run of each test, and the mean average of the two. Starting test was counterbalanced between participants, as described in the Methods.

Further details concerning these and other technical aspects of the test are given in the Supplemental Material.

### Measurements of Luminance Sensitivity

Differential light sensitivity (DLS) was measured at each stimulus location. DLS is defined as smallest detectable difference in luminance, *ΔL*, between the target, *L_targ_*, and the background, *L_B_*. The value of *L_B_*was fixed at 10 cd/m^2^. The value of *ΔL* varied trial by-trial from 0.08 to 225.00 cd/m^2^, as determined by the ZEST algorithm, to find the smallest value of *ΔL* that could be reliably detected on 50% of trials: *ΔL_jnd_*.

Following standard perimetric convention,^50^^–^^52^ DLS values were reported in terms of the amount of signal attenuation on an inverted log scale:

(1) $$DLS_{dB}=10log_{10}\left(\frac{\Delta L_{max}}{\Delta L_{jnd}}\right),$$

where *ΔL_max_* is the greatest displayable stimulus pedestal. For the present test *ΔL_max_ =* 225 cd/m^2^. Therefore, DLS varied between 0 and 34 dB, with higher values indicating greater sensitivity.

For ease of comparison, the values from the HFA were converted into the same decibel scale as Eyecatcher. This was achieved by converting the dB values from the HFA into linear (cd/m^2^) units and substituting them for *ΔL_jnd_* in Eq. 1, thus:

(2) $$\Delta L_{jnd}^{'}=\Delta L_{max}^{'}10^{-DL{S^{'}}_{dB}/10},$$

where $\Delta L_{max}^{'}$ is the greatest displayable stimulus pedestal for the HFA (3183.1 cd/m^2^), and$\;DLS_{dB}^{'}$ is the raw output from the HFA (pointwise sensitivity estimates). After rescaling, any negative DLS_dB_ values (i.e., values for which the smallest detectable value was more intense than the maximum intensity of Eyecatcher) were rounded up to zero. In practice, however, this only occurred at the blind spot (i.e., since all participants were normally sighted).

### Catch Trials

Catch trials (mean *n* = 21 per test) were randomly interleaved with test trials. One-half were blank (invisible target) to assess false-positive response rates; one-half were suprathreshold (maximal luminance) to assess false-negative response rates. Catch trials occurred at the same locations as test trials, but were not included in threshold estimates.

### Statistical Analyses

Each Eyecatcher test yielded 46 DLS test scores (44 test points, and an upper/lower blind spot). To convert these values into an overall index of mean sensitivity (MS) the 44 test points were mean averaged, excluding the two blind spot locations. The HFA provided eight additional test points, but for parity these eight points were excluded when comparing sensitivity estimates between the two tests.

95% confidence intervals were computed for all statistics via bootstrapping (*N* = 20,000), using the bias-corrected and accelerated-percentile method.^53^ A nonparametric bootstrapping procedure, similar in principle to a Mann-Whitney *U* test, was also used to evaluate differences in the 95% coefficient of repeatability (CoR_95_) between Eyecatcher and the HFA (see the Supplemental Material).

## Results

### Mean DLS (MS)

Figure 2A shows the distribution of MS across all 128 tests. For Eyecatcher, the group mean ± SD MS value was 19.7 ± 1.8 dB. The results of the HFA were similar (19.8 ± 1.4 dB), and there was no significant difference in paired MS scores between the tests (paired *t*-test; *t*_127_ = –0.86, *P* = 0.390, NS), with individuals who scored higher on the HFA tending to score higher on Eyecatcher also (Pearson correlation of mean MS scores for each observer: *r*_64_ = 0.59, *P* ≪ 0.001; Fig. 2B).

Note that the imperfect correlation between the two measures was largely owing to the intrinsic measurement error within each measure (see Reliability), combined with the limited individual variability within the present cohort (i.e., because all observers were visually normal, and so exhibited similar MS values). Thus, even if we consider the HFA in isolation, the within-participant correlation between repeated runs was only 0.76 (*r*_64_ = 0.76, *P* ≪ 0.001). As shown in Figure 2B, however, there were individual instances where the results of Eyecatcher and the HFA diverged markedly. These instances predominated at lower sensitivities, and tended to be in observers who exhibited increased false-positive/false-negative rates on the HFA (i.e., instances of possible poor compliance, owing to fatigue or general inattentiveness).

Both tests exhibited a small but significant practice effect (Fig. 2C), with MS increasing between runs one and two by 0.36 dB with Eyecatcher (paired *t*-test; *t*_63_ = 2.69, *P* = 0.009), and by 0.50 dB with the HFA (*t*_63_ = –4.11, *P* < 0.001).

### Distribution of Sensitivity Across the Visual Field

**Figure 3.**
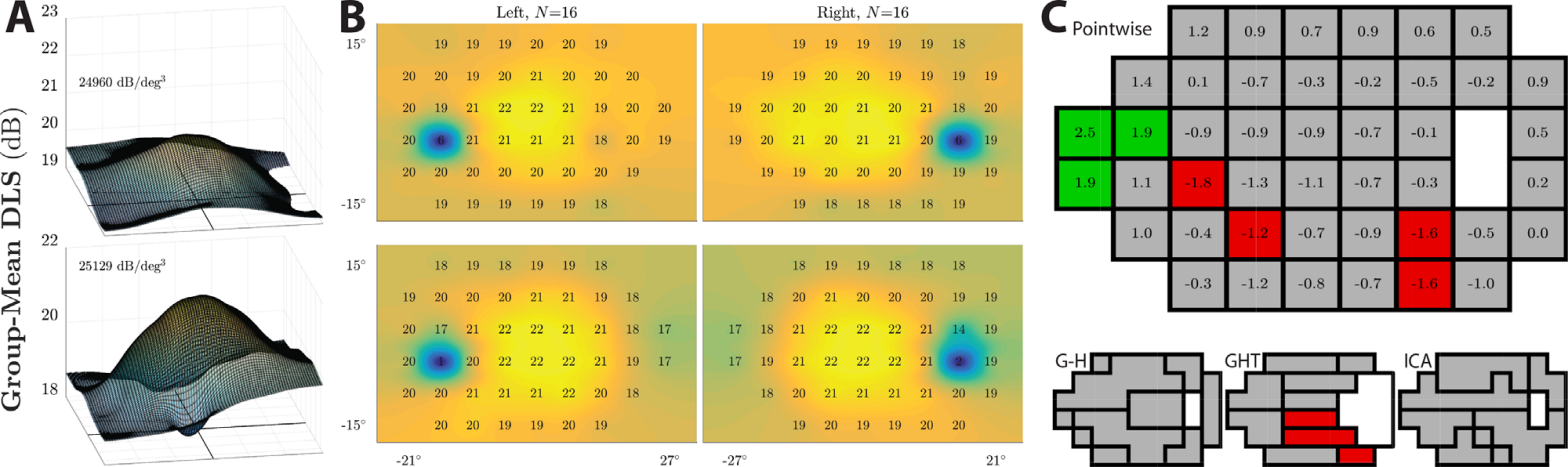
Distribution of pointwise visual sensitivity (DLS) estimates across the visual field. (A) Three dimensional “Hill of Vision” plots for the new test (top) and the HFA (bottom). Surfaces fitted using spring-regularized nearest-neighbor interpolation, and then smoothed using a moving-average rectangular filter. The two blind spot locations were excluded from fits. A top-down view of these hills is given in B. (B) Group mean DLS values for each eye (columns) and test (rows). (C) Differences in DLS values between Eyecatcher and HFA (DLS_Eyecatcher_ – DLS_HFA_), shown for individual pointwise locations (top) and for subregions identified using three popular clustering algorithms (bottom: Garway-Heath map, glaucoma hemifield test map, independent components analysis map; for details see Boden et al^54^). Shading indicates bootstrapped significance tests (red: HFA yielded higher DLS; green: Eyecatcher yielded higher DLS; grey: no significant difference; α = 0.01, Bonferroni corrected).

Eyecatcher was able to measure normal variations in visual sensitivity, with gradations in sensitivity evident between paracentral and peripheral test locations (the “Hill of Vision”; Fig. 3A and Fig. 3B). Furthermore, Eyecatcher exhibited enough spatiotemporal specificity to isolate the physiologic blind spot (Fig. 3B). Thus, sensitivity estimates at 〈±15°, –3°〉 were significantly lower than at any of the surrounding locations (eight paired *t*-tests; all *t*_63_ < –19.7, all *P* « 0.001). Finally, Figure 3C compares sensitivity estimates for Eyecatcher and the HFA at each point on the test grid, and also for various subregions specified by three published visual field maps.^54^ The majority of locations exhibit no significant difference (paired *t*-test; *P* > 0.01; Bonferroni corrected), but nasal DLS values were ∼2 dB higher for Eyecatcher, while some points in the inferior hemifield were 1 to 2 dB lower.

### Reliability (Test–Retest Repeatability)

**Figure 4.**
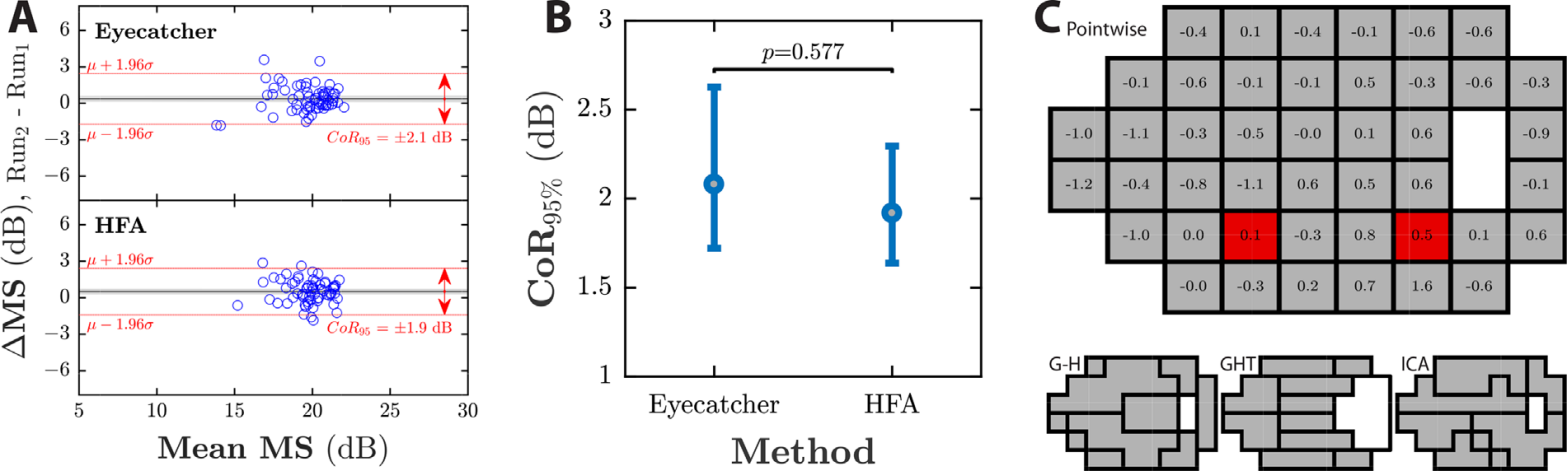
Test–retest repeatability. (A) Bland-Altman plots of MS. Grey shaded regions show 95% confidence intervals around the mean difference. Dashed red lines indicate the 95% limits of agreement (µ ± CoR_95_). (B) Comparison of CoR_95_ values for MS. Error bars indicate bootstrapped 95% confidence intervals. (C) Differences in CoR_95_ values between Eyecatcher and HFA (CoR_Eyecatcher_ – CoR_HFA_), shown in the same format as Figure 3C (NB: positive numbers indicate HFA more reliable).

Bland-Altman analyses^55^ were used to assess the reliability of the MS estimates. The CoR_95_ was ±2.08 dB for Eyecatcher, versus ±1.92 dB for the HFA (Fig. 4A). This difference was not statistically significant (*P* = 0.585; assessed using the bootstrapping procedure described in the Methods), indicating that Eyecatcher is as reliable as the HFA.

To assess whether reliability varied across the visual field, this procedure was repeated for individual DLS estimates at each test location/subregion (Fig. 4C). The CoR_95_ values for Eyecatcher and the HFA differed significantly at two locations, with a mean absolute difference of 0.49 dB, again indicating that the two tests are similarly reliable.

### Test Duration

**Figure 5.**
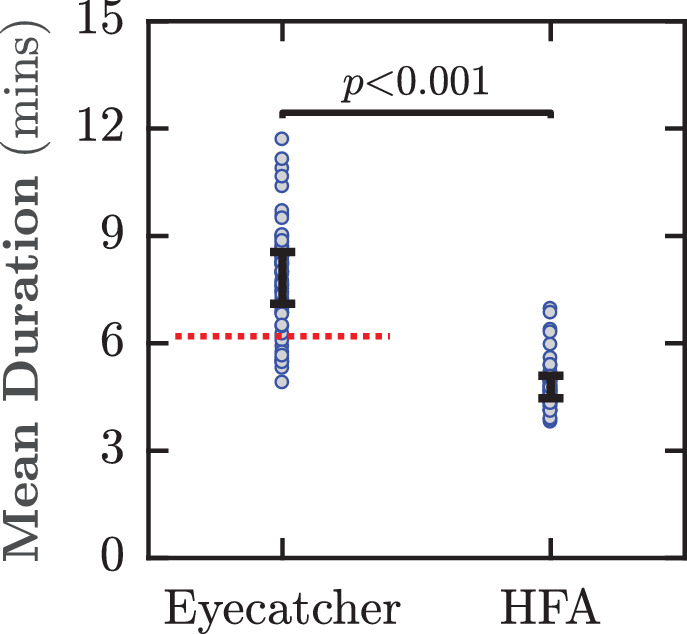
Mean test durations with 95% confidence intervals. The horizontal dashed red line indicates Eyecatcher data from a subsequent follow-up test, in which a more appropriate starting prior was used (see the Supplemental Material).

The mean test duration for Eyecatcher was 7.8 minutes (Fig. 5). This was significantly longer than the HFA duration of 4.8 minutes (paired *t*-tests; *t*_31_ = 28.70, *P* < 0.001). Subsequent follow-up tests indicated that Eyecatcher test durations could be reduced to 6.2 minutes by improving key parameters of the thresholding algorithms (see Supplemental Material), although that still remains significantly slower than the HFA (one-sample *t*-test; t_63_ = –16.75, *P* ≪ 0.001).

### Error Rates

**Figure 6.**
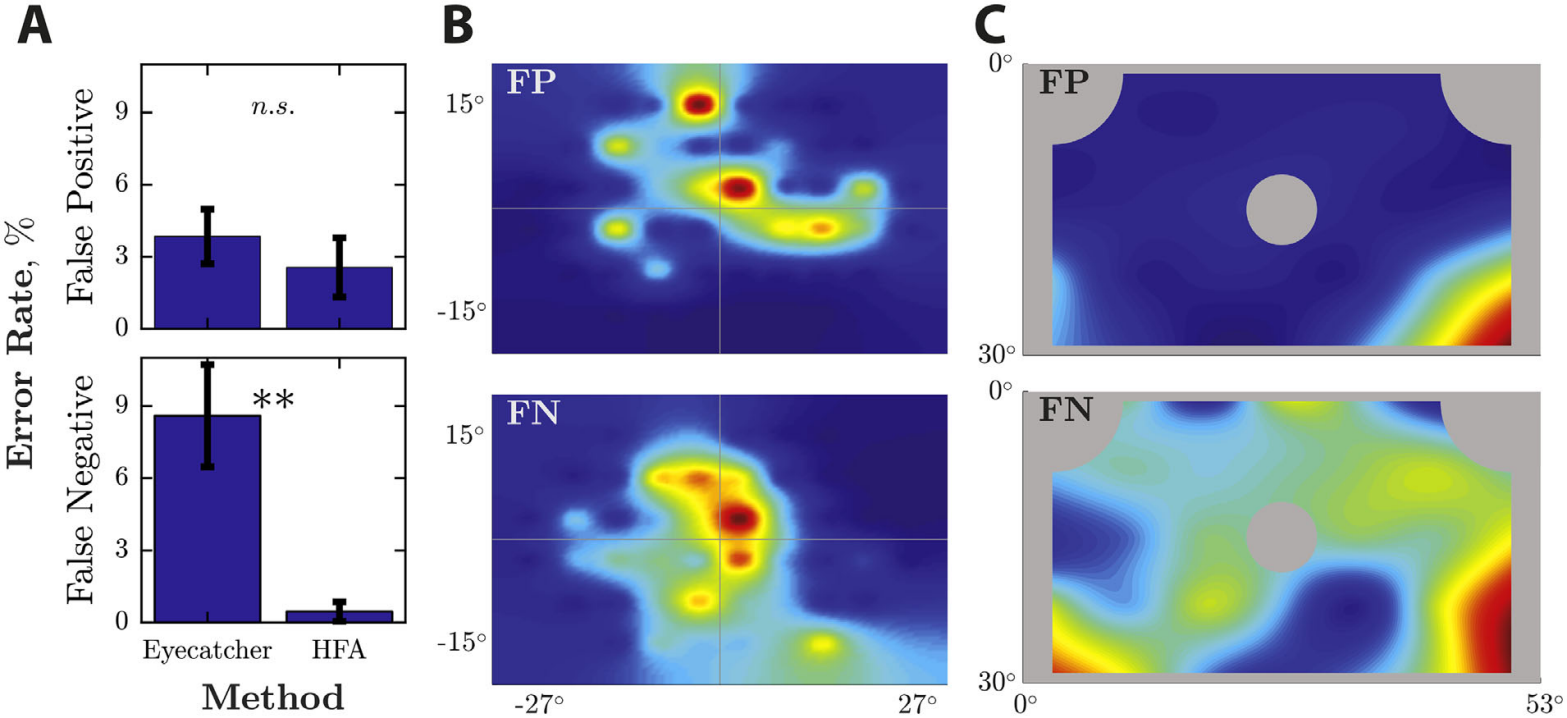
Response error rates. (A) Mean (95% CI) error rates for false-positive (top) and false-negative (bottom) responses. (B) Distribution of errors, relative to the current point of fixation (i.e., in visual-field coordinates). (C) Distribution of errors, in absolute display screen coordinates (NB: the grey areas indicate areas of the screen in which stimuli were prohibited from ever being presented).

The mean error rates are shown in Figure 6A. For Eyecatcher, the false-positive rate (responding to an invisible stimulus) was 2.8%. This rate did not differ significantly from the HFA rate of 2.6% (*t*_63_ = 0.22, *P* = 0.830, NS). However, the false-negative rate (failure to respond to a suprathreshold stimulus) was 7.3%, which was significantly greater than the HFA rate of 0.5% (*t*_63_ = 4.90, *P* < 0.001) (see Supplemental Material for further analyses). To assess how errors were spatially distributed, Figure 6B and Figure 6C show errors as a function of stimulus location. The pattern is clear: classification errors occurred predominantly when targets were located either (i) close to the current point of fixation (Fig. 6B) and/or (ii) in the lower corners of the screen (Fig. 6C).

### Normative 95% Population Limits

**Figure 7.**
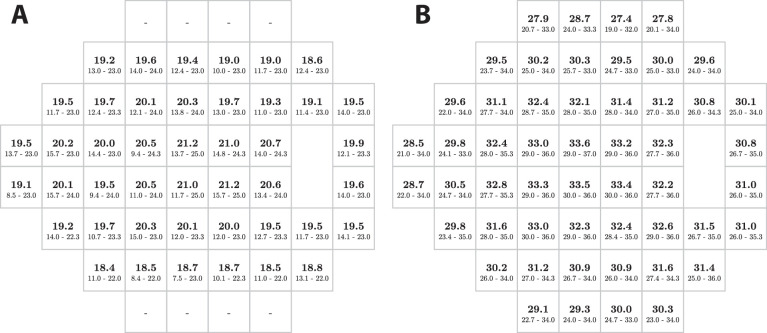
Normative visual field value (DLS in dB) for young, normally sighted adults, as assessed using (A) Eyecatcher, and (B) the HFA. In both cases, figures in bold show the mean estimated sensitivity across all 128 tests. Figures below show the 2.5th and 97.5th percentiles. All tests were converted to right eye format. Dashes indicate locations on the 24-2 test that were omitted owing to technical constraints (see the Methods). HFA data are presented in their original dB units, and so are not directly comparable to Eyecatcher data, but are included here for completeness (see Fig. 3B for scaled values).

Figure 7 shows MS values and 95% population limits for Eyecatcher and the HFA.

## Discussion

This preliminary study evaluated the feasibility of an open-source eye-movement perimeter (Eyecatcher) in young, normally sighted adults. Eyecatcher is a buttonless analog to SAP, which uses an eye-tracker to (i) position stimuli on an LCD screen, relative to the current point of fixation, and (ii) evaluate eye-movement responses. Unlike previously published versions of Eyecatcher, which performed suprathreshold screening only,^38^^,^^59^ here we adapted it to estimate sensitivity thresholds using the popular ZEST thresholding algorithm.^30^^–^^33^

Overall, the results were encouraging. In young, normally sighted adults, Eyecatcher was able to replicate the output of a gold-standard (HFA) perimeter, with no significant difference in MS, and similar test–retest reliability. From a practical point of view, it was also encouraging that participants were able to perform the test without any prior practice or detailed instructions. If it can be shown to operate robustly in patients, such a test might be particularly well-suited for individuals whose gaze or head cannot be fixed, or who are unable to make a taught/button press response (e.g., infants, or stroke patients). Given that it only requires widely available commercial hardware, a test such as Eyecatcher could also be adapted to provide visual field assessments in locations where access to specialized ophthalmic equipment is limited, such as in community centers or developing countries.

### Limitations and Future Work

The primary limitation of the present study is the absence of clinical data from patients or older adults. We have demonstrated previously that glaucoma patients and age-similar controls are capable of performing a shorter, tablet-based, suprathreshold version of Eyecatcher, intended to be used as a triage measure in busy clinical settings.^38^^,^^59^ However, it remains to be seen how well the present, threshold version of Eyecatcher performs in patients, particularly since the present test was relatively slow (discussed elsewhere in this article), and test durations are liable to be further elevated in clinical populations. It is encouraging though that Eyecatcher was capable of detecting normal changes in sensitivity across the normal visual field, and had sufficient spatial resolution to isolate the blind-spot from surrounding points on a 24-2 test grid (i.e., ±6° horizontal, ±6° vertical). This suggests that Eyecatcher may be capable of detecting even mild and relatively localized visual field defects.

There are also a number of limitations with the test itself. We consider each in turn.

#### Eccentricity

The operating range of our eye tracker meant that gaze could only be tracked within a region ±27° (horizontal) by ±15° (vertical). This region is sufficient to replicate the majority of the standard 24-2 perimetry grid, but precludes measurements farther into the periphery. If required, more peripheral measurements could be achieved by combining multiple cameras to track wide angle (±60°) eye movements, as demonstrated recently by Pratesi et al.^56^ Alternatively, in the longer term, head-mounted displays with integrated eye tracking may provide a cheaper and more robust solution. These provide high-quality (e.g., OLED) wide-field displays, and easy control over ambient lighting. A number of such devices are commercially available (e.g., the Fove0, or HTC Vive Eye Pro).

#### Testability

During the present study—and including unreported pilot data—9 in 113 individuals (8%) were unable to complete Eyecatcher assessment. All of these failures were due to the eye not being tracked reliably by the eye tracker. The exact cause(s) of these eye-tracking failures is currently unknown. However, risk factors may include ethnicity^57^ (5 of the 9 test failures involved participants of African descent, whereas the vast majority of the 104 successful cases were Caucasian or East Asian), and/or the wearing of strong optical correction (2 of the 9 test failures involved participants wearing ≥|6| diopter glasses, whereas none of the 104 successful cases did). Data collected subsequently^59^ from 77 patients with glaucoma indicated a similar failure rate (9%), although there refractive correction did not seem to be a substantive confound (the vast majority of patients wore glasses, including strong multifocal prescriptions), and most failures appeared to be related to recent surgical interventions (e.g., cataract removal). Taken together, it is clear that eye-tracking failures would substantively limit any ophthalmic applications, and completion rates will need to be improved for eye-movement perimetry to become viable. One solution may be to simply use more sophisticated hardware. Thus, the present study used a relatively old and extremely inexpensive eye-tracker (Tobii EyeX: Released 2014 for ∼$100). Newer, more advanced eye-tracking devices now exist with improved optics and greater spatiotemporal precision, which might be expected to perform more robustly. Alternatively, it may be that there will always be a subset of individuals whose eyes cannot be tracked with sufficient reliability, in which case eye-movement perimeters might be best targeted at particular populations where the eye-tracking technology works best and where the need for SAP alternatives is greatest (e.g., young children with impairments of the retina or optic pathways).

#### Test Duration

Test durations were, on average, 3 minutes longer for Eyecatcher than the HFA, despite the HFA including eight additional test points. Follow-up tests carried out subsequently indicated that this difference could be decreased to less than 2 minutes through improvements to the test algorithms. However, it is likely that Eyecatcher may always be somewhat slower than a button press procedure, owing to the need for additional calibration, refixation, and stabilization trials (mean *N* = 94 per test). It is worth noting, however, that many participants nevertheless reported, anecdotally, that the eye-tracking procedure was more comfortable, despite the longer test duration, owing to the absence of a chin rest or fixation cross. This advantage is important if Eyecatcher is to be used with young or infirm patients.

#### Screen Calibration

To precisely control the luminance of the stimulus and the background, the monitor in the present study underwent detailed photometric calibration, using specialist equipment (see Supplemental Material). This is not a problem for a well-equipped research laboratory, but could be a substantive practical impediment for the use of a test such as Eyecatcher more generally. For this reason, we chose for the present study to use a screen that contains its own inbuilt photometer (EIZO CG277). The inbuilt photometer was not used in the present work. However, we have demonstrated subsequently that measurements from this inbuilt photometer can be substituted for a formal photometric calibration, without any appreciable loss of accuracy or reliability in visual field measurements.^58^ Furthermore, we have made the necessary Matlab code for controlling the photometer available online (https://github.com/petejonze/myEIZOSensor), allowing calibration to be performed automatically (e.g., overnight or before the first test of each day). Finally, we have also shown that this monitor (which comes with a factory-calibrated certificate of uniformity) remains photometrically stable over its spatial extent for at least 20 months.^58^ Taken together, this makes the prospect of a simple plug-and-play perimeter—one that combines open source software with ordinary, commercially available hardware—a genuine, pragmatic possibility.

## Conclusions

(i) There was no significant difference in mean luminance sensitivity (MS) between Eyecatcher (M = 19.7 dB; SD = 1.8 dB) and the gold standard HFA (M = 19.8 dB; SD = 1.4 dB).
(ii) Eyecatcher was also able to detect spatial differences in sensitivity across the normal visual field (the “Hill of Vision”), and could differentiate the physiological blind spot from surrounding points (*P* ≪ 0.001).
(iii) There was no difference in reliability (test–retest repeatability) between Eyecatcher (CoR_95_ = ±2.08 dB) and the HFA (CoR_95_ = ±1.92 dB).
(iv) Eyecatcher was slower than the HFA (7.8 minutes vs. 4.8 minutes), although it is estimated that some of this the difference could be reduced through improvements to the test algorithms. Nevertheless, participants reported informally that the new procedure was more comfortable than methods relying on a fixed head position on a chin rest.
(v) We conclude that remote eye tracking can be used to perform hands-free visual field assessments in normal seeing adults, using off-the-shelf commercial hardware and the free software presented here. Normative values are provided, and further refinements are suggested to make the test more clinically robust.

## Supplementary Material

Supplement 1
