# Supplementary material for: An Open-source Static Threshold Perimetry Test Using Remote Eye-tracking (Eyecatcher): Description, Validation, and Preliminary Normative Data
Source: Transl Vis Sci Technol. 2020 Jul 13;9(8):18. doi: 10.1167/tvst.9.8.18 (PMC7422828; doi:10.1167/tvst.9.8.18)
Supplement: Supplement 1 [file tvst-9-8-18_s001.pdf]

Supplemental Material

An open-source static threshold perimetry test using remote eye-tracking (Eyecatcher): Description, validation, and preliminary normative data.

Pete R. Jones[1,2,3]

[1]*University College London (UCL), Institute of Ophthalmology, 11-43 Bath St, London, UK, EC1V 9EL; p.r.jones@ucl.ac.uk*

[2]*NIHR Moorfields Biomedical Research Centre, 162 City Road, London, UK, EC1V 2PD*

[3]*City, University of London, School of Health Sciences, Division of Optometry and Visual Sciences, London, UK, EC1V 0HB; peter.jones@city.ac.uk*

S1. DOWNLOADING SOFTWARE

S1.1. Download location

The MATLAB source code required to run the reported Eyecatcher test can be downloaded from the project homepage: `https://github.com/petejonze/Eyecatcher`. Please note: the code is provided as-is, and non-trivial modifications may be required to install and run it. We hope in future to develop a simple executable version of the test, but at the time of writing this should be considered 'research grade' code.

S1.2. License

The code is available under a GNU GPL v3.0 license, which allows the software to be freely used, modified, and distributed. Any modifications to, or software including this code must also be made available under the GNU GPL v3.0 license.

S1.3. Programming language & operating system

The code is written primarily in MATLAB, and will work on any platform that supports MATLAB 2012 or newer (including Mac OS X, Linux, and Microsoft Windows). Note, however, that this code requires input from an eye-tracker, and some manufacturers only provide drivers for selected operating systems. For example, the Tobii EyeX eye-tracker only supports Windows. The code has been designed primarily using Tobii EyeX eye-trackers, but can be easily modified to work with input from other devices, and example code for other popular devices is provided (Tobii Pro series, Eyelink 1000, SMI Red).

S1.4. Additional dependencies

The code requires Psychtoolbox v3.0 (`http://psychtoolbox.org`).

S2. TECHNICAL DETAILS

S2.1. Choice of software/hardware

Graphics card. An Nvidia Quadro K620 graphics card was used for threshold testing, because it supports 10-bit luminance control (when connected to a 10-bit monitor via a DisplayPort adapter). Other 10-bit graphics cards, such as the AMD FirePro series (Advanced Micro Devices, Inc.; Sunnyvale, California, USA), are also available, but were not tested. Unlike with a standard 8-bit display, the smallest luminance difference on a 10-bit display is subliminal for human observers. This gives more precise stimulus control, and also means that differences in display uniformity can be corrected without visible steps in background color. An alternative, cheaper solution is to use bit-stealing[?] (chromatic dithering) to simulate 10.7-bit luminance on a standard 8-bit display. During initial piloting, no substantive difference in test results was observed between native 10-bit and simulated 10.7 bit performance. However, given falling hardware costs, the savings of using an 8-bit display are relatively small. Conversely, bit-stealing introduces visible chromatic artifacts, complicates the underlying code, and prevents chromatic feedback from being interleaved with test stimuli (i.e., since it only supports monochromatic graphics).

LCD screen. Testing was performed using an EIZO CG277 monitor. The size of this monitor is 59.7 x 33.6 cm (2560 x 1440 pixel), and it was viewed at a distance of approximately 60 cm (∴, 52.9° x 31.3° visual angle). This display device was chosen as it has a certified level of spatial uniformity ($\Delta E \leq 3$), a wide intensity range (0 – 300 cd/m^2, though manually limited in the present test to 0 – 245 cd/m^2), a reasonably fast response time (6 ms; gray-to-gray), an IPS panel with a reasonably wide viewing angle (half-angle = $\pm 89°$), a fast warm-up time ($\sim$7 minutes), and supports native 10-bit luminance. This screen was also of particular interest as it has an integrated photometer and ambient light sensor, which can potentially be used to calibrate the screen (*study ongoing*). The screen was VESA-mounted on an Ergontron extendable arm for comfortable viewing and easy repositioning (Ergrotron Inc., Eagan, Minnesota, USA). During piloting, a 30 inch Samsung SyncMaster 305T LCD Monitor (Samsung Electronics Co. Ltd., Seoul, South Korea) was also used. This also appeared to provide acceptable results. However, it was not used during testing as the 305T does not support 10-bit luminance, does not contain an integrated photometer, and is generally lower-spec.

Eye-tracker. A Tobii EyeX eye-tracker was used during testing because: it supports monocular tracking, operates remotely without any observer attachments, can estimate viewing distance, and has a short operating distance ($\sim$60 cm – allowing even a reasonably small monitor to support a 24-2 test grid). The Tobii EyeX device was also of particular interest, as, with a retail price of $\sim\$100$, it provides a lower bound on what might be achieved given a more expensive, 'research grade' eye-tracker. The Tobii EyeX, however, may not appropriate for general use due to: (i) its restrictive terms of use that discourage 'research' or 'medical' purposes (beyond proof-of-concept piloting), and (ii) the emergence of more advanced eye-tracking devices. For example, since testing was complete, Tobii have launched an 'X3-120' device, which has over twice the sampling rate of the EyeX. Other remote eye-tracking devices, such as the SMI Red (Sensomotoric Instruments GmbH, Teltow, Germany), the Eyelink 1000 Plus (SR Research Ltd, Oakville, Canada), or the Livetrack FM (Cambridge Research Systems, Rochester, UK) might also be capable of supporting the reported visual-field test, but have not be tested.

S2.2. Key differences between the Eyecatcher procedure and traditional automated static threshold perimetry [ASTP] methods

As with the HFA, targets in Eyecatcher were 0.43° diameter (Goldmann III) circles of variable luminance. These were presented on a 24-2 grid, against a 10 cd/m^2 white background. However, unlike with the HFA:

1. Participants responded by making an eye-movement towards the target location, rather than by pressing a button. This is an intuitive response, which occurs spontaneously even in newborn infants[?]. Participants were instructed to “look at anything that appears on the screen” and were told, “the test will be completed most quickly if you only move your eyes when you see something”.
2. Participants were not required to maintain fixation on a central cue. Instead, target stimuli were presented relative to the current point of fixation: wherever the participant was fixating at trial onset. Typically, this would be the target location from the previous trial. In practice, this lead to a number of practical challenges, and algorithms were developed that automatically: (i) waited for steady fixation before presenting a target, and presented a ‘stabilization’ cue after 500 msec if required; (ii) presented ‘refixation’ cues to shift gaze position when a target would otherwise fall outside the screen area; (iii) avoided placing targets near the center of the screen (due to a widespread bias to fixate there[?] in the absence of a stimulus); (iv) avoided placing targets in areas of the screen that exhibited poor tracking accuracy/precision. Further details concerning stimulus placement are given below.
3. Participants sat normally on a standard office chair, and head location was not constrained. The eye-tracker remotely tracked eyeball location, and so was able to stabilize gaze location, independent of minor head movements. In addition, the distance of the participant’s eyeball was used to dynamically scale the size of the stimulus on the screen, to ensure a constant stimulus size on the retina (invariant of viewing distance).
4. The four test-points from the top and bottom of the standard 24-2 grid were omitted. This was due to technical limitations – current eye-trackers tend to have limited vertical range, and often exhibit poor precision and systematic inaccuracies in the vertical extremities[?]. The omission of these points is unlikely to have affected the present findings. However, some of the omitted points are informative for certain clinical populations (e.g., some glaucomatous eyes exhibit pronounced deterioration in the upper extremity[??]). In principle, these test-points could be reintroduced, either through improvements in hardware, or using additional ‘refixation’ trials to shift the patient’s gaze to the extremities of the screen.
5. As the HFA’s (‘SITA-standard’) thresholding algorithm is proprietary technology, the qualitatively similar ZEST algorithm[????] was used to adapt stimuli and determine detection thresholds. The prior was a bimodal probability density function, constructed by combining normative data for healthy and glaucomatous eyes, as per Ref∼[**?**]. The likelihood function was a cumulative Gaussian, with a fixed slope of $\sigma = 1.25$, and a variable mean of $\mu = \langle 0, 1, 2, \ldots, 34\rangle$ (i.e., target levels were uniformly distributed on a log-scale, and were adaptively varied in dB integer steps). The growth pattern is given in Figure 2C of the Main Manuscript. The starting guess was determined by normative data[?] for the initial points, and by the arithmetic mean of adjacent estimates thereafter (though see *S3.1. Luminance-corrected eye-tracking procedure: Correcting for a technical error in calculation of* ΔL). A dynamic termination criterion was used[??], in which the spread of the estimated posterior function was required to have a standard deviation of $\sigma \leq 1.5$ dB.
6. In addition to trials in which test-points were presented (Mean N = 256 per test), further ‘ancillary’ trials were interleaved throughout testing (Mean N = 94 per test). These consisted of: (i) suprathreshold refixation trials (Mean N = 51), to allow eccentric stimulus placement; (iii) suprathreshold stabilization trials (Mean N = 4), when participants were making excessive eye-movements prior to stimulus onset (iii) suprathreshold calibration trials (Mean N = 18), to calibrate the eye-tracker; (iv) suprathreshold catch trials (Mean N = 10), to evaluate false-negative response rates; (v) subthreshold (blank) catch trials (Mean N = 11), to evaluate false-positive response rates. The 21 catch trials were used to evaluate error rates, but could be omitted from clinical tests (e.g., as per SITA FAST).
7. Stimuli were presented on a planar surface (a ‘tangent screen’), not on an arc concentric

with eye (as with dome perimeters, such as the HFA). Equal distances on a plane do not correspond with equal angles in the eye[?]. The shape and size of stimuli were therefore warped in software, to ensure constant shape/location on the retina (invariant of eccentricity).

8. To remove stimulus edge-effects (a potential detection artefact), a 2-D Gaussian low-pass filter was applied to the stimuli. This ensured that stimuli were contrast-modulated smoothly at their edges. Filtering was performed through convolution, using a 2-D finite impulse response (FIR) filter, and a rotationally symmetric Gaussian kernel of size 15x15 px and standard deviation 2.85 px.
9. Once all test points were complete, any suspect estimates were retested. Suspect estimates were identified as follows. For each test location, the difference was computed between estimated DLS, and expected DLS given prior normative data. These differences were converted to Z-scores, based on the observed distribution of differences across all of the 44 test points for that individual. Any differences > 2 Z-scores in magnitude was classified as an outlier, and retested. Thus, a single highly deviant point would be retested, but if all points were consistently higher/lower than expected then no points would be retested. In practice, the median number of points retested was two.

S2.3. Stimulus selection (ZEST)

The algorithm for selecting target stimuli is shown schematically in **Figure S1**. In short, test trials were uniformly randomly selected from the current 'wave' of test locations. Waves proceeded in sequence from 1 to 4, in the manner shown in Figure 1C of the Main Manuscript. In addition to test trials, blind-spot trials and catch trials were randomly interleaved throughout testing.

If a selected target location could not be displayed (i.e., fell outside the valid test area of the screen), then a new location was selected. If, after four attempts, no valid target location had been generated, then a 'refixation' trial was used to manually move the participants view to a pseudo-random location, such that the current target location could be presented on the following trial.

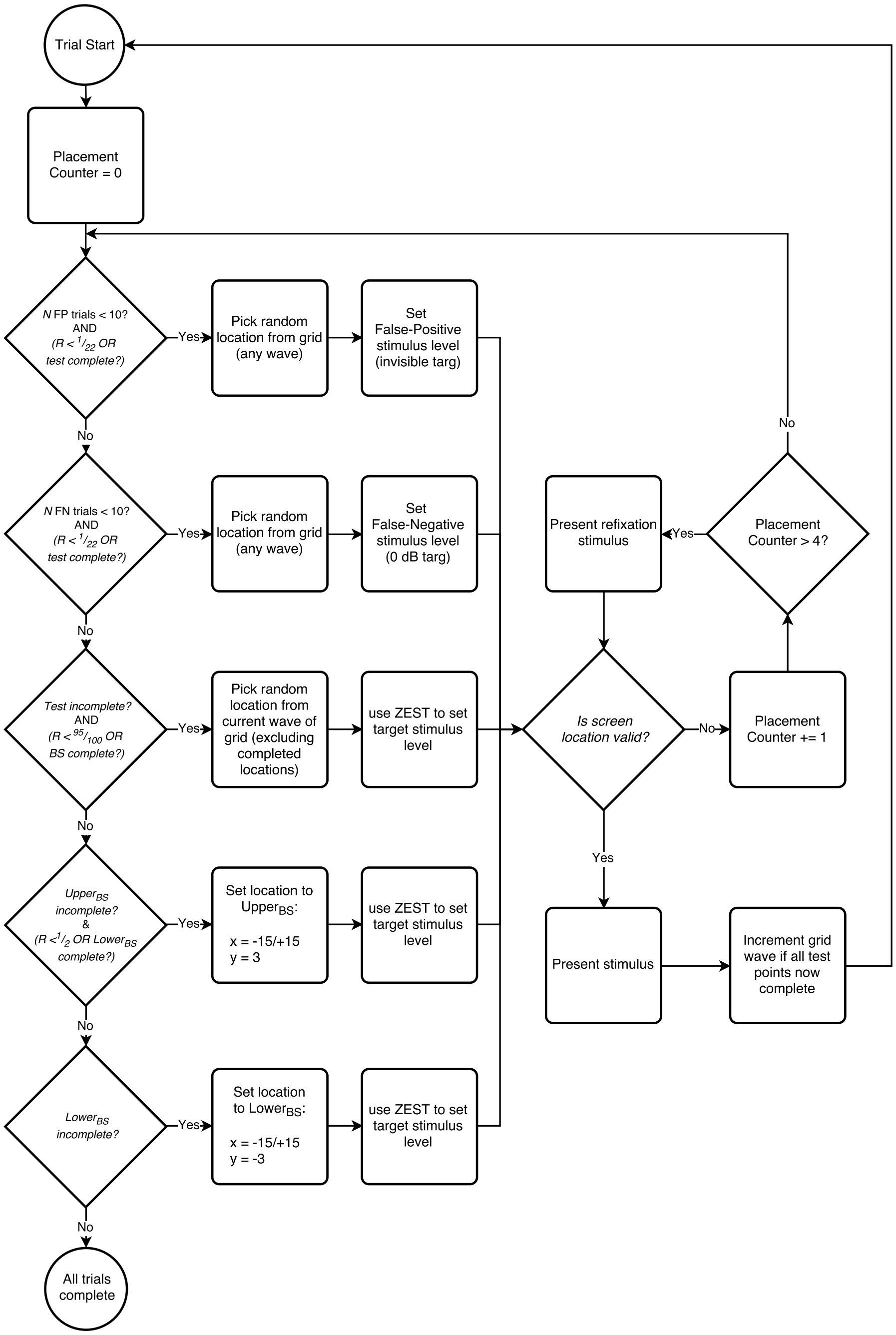

Fig S1. Flowchart showing how the target stimulus was selected on each trial.

S2.4. Stimulus placement

The valid test regions of the display screen are shown in **Figure S2** (areas not shaded red). In accordance with perimetric standards[?], target edges were not permitted to fall within two stimulus-diameters of the screen edge. In addition, target centroids could not fall within:

1. 3 degrees of the (left/right) sides of the screen
2. 7.2 degrees of the upper-left/right corner of the screen
3. 3.6 degrees of the screen center of the screen

Since classification errors occurred most frequently in the bottom corners of the screen, future iterations of the test will likely extend criterion (2) to these regions too.

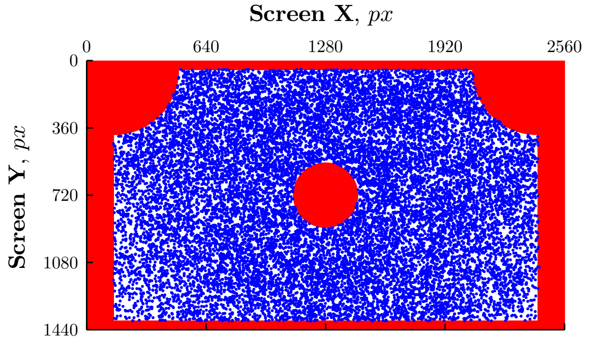

Fig S2. Target placement. Valid/invalid areas of the screen are shown in grey/red, respectively. Blue circles indicate stimulus locations in all individual trials during the 128 (2x64) tests reported in the main manuscript.

S2.5. Eye-tracker calibration

Gaze calibration overview. To map estimated gaze locations (x/y screen coordinates, in pixels) to their true values, participants completed a calibration procedure in which they were directed to sequentially fixate nine highly visible/salient targets (**Figure S3A**). Standard algebraic regression was then used to find the second-order polynomial surface that best predicts the 'true' gaze coordinates, given the observed gaze coordinates (minimizing least-square Euclidean error). This calibration procedure was carried out before the first trial, and then again: (i) every 400 trials; (ii) if the false negative response rate exceeded 20% (after a minimum of five suprathreshold catch trials).

Two precautions were taken to ensure good quality calibrations. Firstly, as illustrated in **Figure S3B**, regression fits were made twice, with statistical outliers from the first fit excluded from the second. Fits were performed recursively in this way in order to avoid the calibration being biased by erroneous measurements (e.g., because the observer fixating the wrong location, or due to measurement error – for example, due to flickery contact or eye-blink artefacts). Secondly, as detailed in the next section, a log-likelihood classifier was used during the presentation phase to ensure that observers fixated each point. Points that were not deemed to have been fixated (within three seconds) were repeated, until either a successful fixation was detected, or until âĂŞ in extreme cases – the test was manually aborted by the experimenter.

Ensuring that gaze-calibration targets were fixated. A log-likelihood classifier[?] was used to ensure that observers fixated calibration targets (non-fixated calibration targets were presented again). This classifier worked as follows:

Two fixation distributions were hypothesized: A 'Miss' distribution, which predicted where gaze coordinates would fall when not fixating the target; and a 'Hit' distribution, which predicted

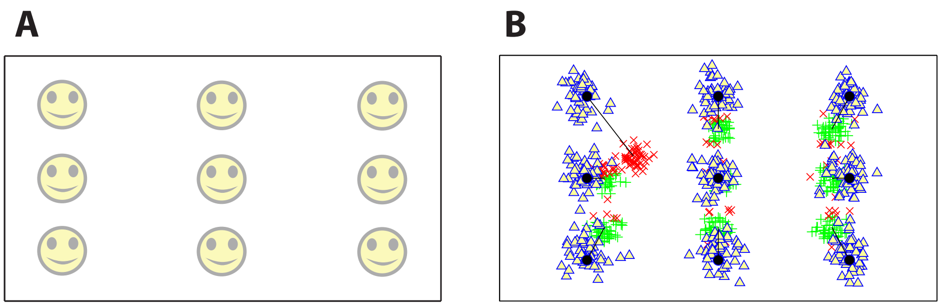

Fig S3. Eye-tracker gaze calibration. **(A)** Calibration locations and target. Targets were positioned at nine locations across the screen, and were presented sequentially, in random order. **(B)** Surface fitting, showing: calibration target locations (black filled circles); raw gaze measurements used for calibration (green plus signs); statistical outliers automatically excluded from the calibration (red crosses). Blue triangles show new gaze coordinates, generated at the same nine average locations as the initial measurements, after applying the fitted calibration. Note that the calibration procedure described here occurred after, and in addition to, any calibration using the eye-tracker's own proprietary calibration routines.

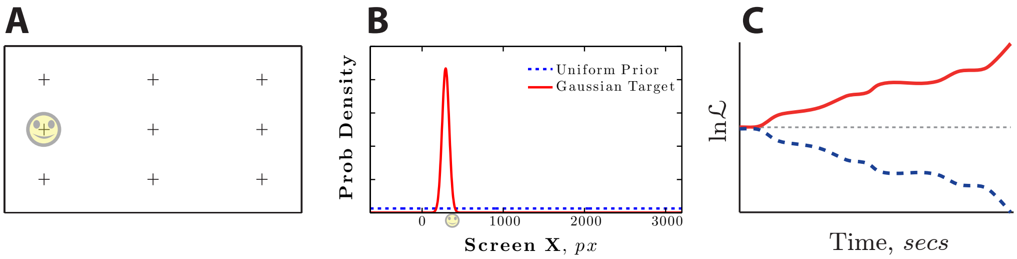

Fig S4. Classification of gaze during eye-tracker calibration. **(A)** Target location. **(B)** Hypothesized distributions of eye-gaze, given target fixation (solid red) or random eye-movements (dashed blue). **(C)** Example classifier 'random walk', as evidence is accumulated over time.

where gaze coordinates would fall when fixating the target. As shown in **Figure S4B**, each distribution only used the horizontal (x-coordinate) gaze data, as these were found to be more reliable than uncalibrated vertical (y-coordinate) gaze estimates.

For each gaze-coordinate returned by the eye-tracker, x_j, the classifier computed the log-likelihood of each distribution being true, given the observed gaze data. At each timepoint, the sum of the log-likelihoods, $\mathcal{LL}$, for each distribution was computed:

$$\mathcal{LL} = \ln\mathcal{L} = \sum_{j=1}^{N} \ln\Big[p(x_j)\Big] \qquad \textbf{S1a}$$

The ratio of these two log-likelihoods was the dependent variable that was used to classify the observer's response (**Figure S4C**). Note that the use of logarithms is purely pragmatic: increasing computational efficiency and preventing numerical underflow. Note also that the use of summation presumes that each gaze-estimate is independent. This assumption is incorrect, strictly speaking, but is an acceptable approximation that simplifies the mathematics considerably.

The probability of observing the gaze coordinates given the 'Miss' distribution, $p_{Miss}(x)$, was specified by a continuous uniform probability density function, which extended across the number of pixels in the whole screen, plus an additional ±25% margin:

$$\begin{aligned} p_{\text{Miss}}(x) &= U(x, a, b) \\ &= \frac{1}{b-a}, \end{aligned} \quad \textbf{S1b}$$

where in the present setup, a = -640 and b = 3200 (i.e., monitor width = 2560 px; margin = ±640 px). Thus, it was assumed that an observer who did not fixate the target would look randomly, anywhere on the screen. To prevent spurious ($-\infty$) values, likelihood values for coordinates outside of the range $\langle a, b \rangle$ were changed from 0 to 1E-09.

The probability of observing the gaze coordinates given the 'Hit' distribution, $p_{Hit}(x)$, was specified by primarily by Gaussian probability density function, $\phi(x, \mu, \sigma)$, centered on the target:

$$\begin{aligned} p_{\text{Hit}}(x) &= 0.95\phi(x; \mu, \sigma) + 0.05U(x, a, b) \\ &= \frac{0.95}{\sigma\sqrt{2\pi}} e^{\left(-\frac{(x-\mu)^2}{2\sigma^2}\right)} + \frac{0.05}{b-a}, \end{aligned} \quad \textbf{S1c}$$

where μ is the center of the calibration image location, and σ = 43.75 (one eighth of the calibration image width – this figure was determined by informal piloting, and meant that ~20% of the distribution fell within the circular target). Note that the Gaussian distribution was linearly mixed, 19:1, with a uniform pedestal , to prevent sudden spikes in the value of p_{Hit} due to isolated outliers (e.g., because of technical errors, or eye-blinks). As with the Miss distribution, likelihood values for coordinates outside of the range $\langle a, b \rangle$ were changed from 0 to 1E-09.

To make a classification decision, the difference between the two log-likelihood metric was compared to a static criterion, λ:

$$\text{Resp} \rightarrow \begin{cases} \text{'Hit'}, & if \quad (\mathcal{LL}_{\text{Hit}} - \mathcal{LL}_{\text{Miss}}) > \lambda_{\text{Hit}} \\ \text{'Miss'}, & if \quad (\mathcal{LL}_{\text{Miss}} - \mathcal{LL}_{\text{Hit}}) > \lambda_{\text{Miss}} \\ \text{Wait}, & otherwise \end{cases} \quad \textbf{S2}$$

For the Hit distribution, $\lambda_{Hit} = 800$. For the Miss distribution, $\lambda_{Miss} = \infty$, meaning that the system would in practice wait forever until a Hit was detected. However, if no decision was reached after 3 seconds, then the classifier defaulted to 'Miss'. In future, this simple heuristic could be replaced by a more principled algorithm, once enough normative data has been collected.

In practice, the log-likelihood metric in **Eq S1a** was modified in order to account for the facts that: (i) eye-gaze is not stationary, and (ii) it takes time for observers to move their gaze to the target location. Generally, the classifier is therefore interested only in the most recent gaze samples, and those that occur a given interval after target onset. To reflect these facts, gaze samples were linearly weighted:

$$\mathcal{LL} = \sum_{j=1}^{N} \left(\omega_j \ln\left[p(x_j)\right]\right), \quad \textbf{S3a}$$

where weights, ω_j, were determined in two ways. Firstly, only the last 90 gaze samples were ever considered (i.e., $\omega = 0$, for any samples occurring $\gtrsim$ 1.8 seconds previously). Secondly, samples occurring shortly after target onset were ramped using the following equation, which is shown graphically in **Figure S5**:

$$\omega_j = \min\left(1, \max\left(0, \frac{t_{\text{ramp1}} - t_j}{t_{\text{ramp1}} - t_{\text{ramp2}}}\right)\right), \qquad \textbf{S3b}$$

where t_j is the time of sample x_j in seconds, and t_{ramp1} and t_{ramp2} are the inflection points of the onset ramp, as shown in **Figure S5**. In practice, $t_{ramp1} = t_0 + 0.4$, and $t_{ramp2} = t_0 + 0.8$, where t_0 is the time of target onset, in seconds).

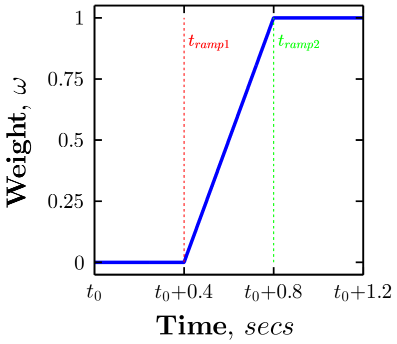

Fig S5. Weighting of incoming gaze-samples, when using the log-likelihood gaze classifier (**Eq S3a**). See body text for details.

Overall, this classifier — which is the same as we have used previously to calibrate gaze coordinates in infants[?] — performed well. However, it may be worth noting that, should a large, systematic error discrepancy exist between actual-versus-estimated gaze coordinates, the classifier may fail to ever register a Hit, thereby preventing the calibration from completing. In principle, this might be resolved by performing a two-step procedure, in which additional calibration targets are initially placed centrally and/or in which the classifier has a larger error tolerance (the σ parameter in **Eq S1c**). Alternatively, operators could in future be given the option to manually override the classifier, if they were confident that the observer was fixating the calibration target.

Additive drift correction (gaze). In addition to the gaze calibration procedure detailed above, an additive drift-correction mechanism was used to maintain gaze-estimation accuracy throughout testing. Immediately after calibration, the error in estimated gaze location was assumed to be $\langle 0, 0 \rangle$. Subsequently, after each trial where the observer was sure to have fixated the target (see below), the error between target location and gaze location was computed. This error was then integrated with the current running total, via a process of weighted-vector-addition. All future measurements were then transposed by this amount.

In practice: (i) only suprathreshold 'attention grabber' trials were used to update the drift-correction factor, and of these, only those subset of 'attention grabber' trials where the observer was classified as having fixated the target; (ii) new offsets were linearly-integrated with the running total, using a weight of 0.15 (i.e., allowing the correction factor to be gradually refined over the course of the experiment); (iii) computed offsets that deviated by a Euclidean distance of more than 6 degrees from the current running total were assumed to be erroneous, and were not integrated with the running total.

Additive drift correction (Z distance). It was observed that estimated distance of the eyeball from the screen was subject to random error between individuals. Thus, viewing distance was

consistently underestimated in some individuals, and overestimated in others. To correct for this, participants were initially instructed to sit at a fixed distance from the screen (60 cm – confirmed by aligning the participant with markings on the wall). The difference between the reported distance (as estimated by the eye-tracker) and 60 cm was computed, and was subtracted from all future measurements. In future, it may be possible to simplify this process, by using a laser range-finder or ultrasonic distance sensor to provide an objective measure of viewing distance.

S2.6. Processing raw eye-tracking data

Incoming eye-tracking data were cleaned/processed in three steps. (1) Outlying values were excluded. (2) Small gaps in data were filled-in using linear interpolation (3) Data were smoothed (low-pass filtered). This steps are visualized in **Figure S6**.

Excluding outliers. Eye-trackers are liable to occasionally report spurious values. This can be due to hardware/measurement error, or because the eyes could not be tracked momentarily (i.e., at which point the device may default to an arbitrary/impossible value, such as '-1'). Such spurious values were identified and replaced with 'blank' values, to be filled by interpolation (see next). In the present test, outlying values were defined as those where either: (i) the eye-tracker self-reported an invalid sample code; (ii) the reported eyeball location was '0 cm'; (iii) the reported gaze location was more than 2000 pixels outside the screen area.

Interpolating missing data. Linear interpolation was used to 'fill-in' small amounts of missing data, such as those caused by poor registration of the eye by the eye-tracker. Missing gaze coordinates were replaced with the arithmetic mean of the two points either side ($\omega \pm 1$). If this failed to yield a valid value (i.e., if one of these data points are themselves missing) then the window size, ω, was progressively increased to $\omega \pm 2$. If this failed (i.e., if there was an extended run of missing values, as with a blink or head-turn), no further interpolation was attempted.

Smoothing. Low-pass smoothing was used to reduce any random error in eye-gaze estimates, arising due to either measurement error or nystagmus. Filtering was performed by computing the running mean ('Moving Average') of incoming gaze coordinates. A running mean is an efficient way to implement a low-pass filter in the time-domain, and resulted in gaze-coordinates that were more consistent over time. However, a wider averaging window introduces inertia into the gaze-data, and also increase lag. In the present test, the size of the window was therefore fixed at a relatively narrow value of ± 2 samples. Note that filtering occurred after internal processing by the eye-tracking device. In the present case, this consisted of a proprietary 'lighting filtering' algorithm, which involved an adaptive filter based on eye-movement velocity.

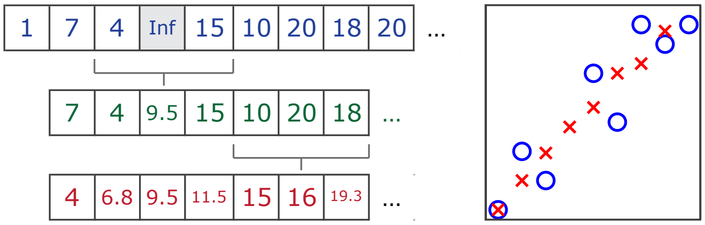

Fig S6. Schema showing how incoming data (1st row/blue circles) were interpolated (2nd row) and smoothed (3rd row/red crosses); here using a window of ± 1 sample. Processing was carried out independently for each gaze coordinate $\langle x, y \rangle$. See body text for details.

S2.7. Eye-movement evaluation (Classifying hits/misses)

To determine whether the participant looked at a target location (a 'Hit'), we used a simple 'hit box' classifier, in which:

$$\text{Resp} \rightarrow \begin{cases} Hit, & if \quad N \text{ gaze estimates fell within a } D^\circ \text{ x } D^\circ \text{ box,} \\ & \quad \text{within } R_{max} \text{ seconds of stimulus onset.} \\ Miss, & otherwise. \end{cases} \quad \textbf{S4}$$

Gaze estimates did not have to be consecutive, so all samples occurring within Rmax seconds were counted, even if some samples strayed outside of the hit box (although, given the brief time period, this was more likely to occur due to nystagmus and/or measurement error, rather than saccadic eye-movements). It was assumed participants would be slower and less accurate at fixating more distant target locations. Therefore, to minimize false-negative responses, the parameters N, D, and R_{max} varied as function of stimulus eccentricity, E, thus:

$$N = 10 - \text{nint}\left(\min\left\{9, \max\left\{0, \frac{(E-5)}{2}\right\}\right\}\right) \quad \textbf{S5a}$$

$$D = 2 + \max\left\{0, \frac{(E-5)}{10}\right\} \quad \textbf{S5b}$$

$$R_{max} = \frac{N}{F_s} + 1.5 \quad \textbf{S5c}$$

where E is the Euclidean distance of the target from the participant's point of fixation at trial onset, in degrees, and where F_s is the sampling rate of the eye-tracker, in Hz. For example, given an eye-tracker with a sampling rate of 50 Hz, a target at $\langle +9^\circ, +9^\circ \rangle$ would be scored as seen (only) if the participant's gaze fell within 2.77° of the target for 6 samples (120 milliseconds), within 1.62 seconds of stimulus onset.

To minimize false-positive responses (e.g., due to random searching of the screen), two additional heuristics were used. Firstly, if participant's gaze deviated outside a ± 8° region, extending linearly from initial gaze location to target location, then the trial was classified as a 'Miss' and immediately aborted. Secondly, if a 'Hit' occurred within Rmin seconds of stimulus offset, then the trial was repeated[i].

$$R_{min} = \frac{N}{F_s} + \frac{1}{3} \quad \textbf{S5d}$$

Note that these algorithms are coarse heuristics based on limited pilot data, and they prioritized simplicity over any formal measure of optimality. Their form and parameterizations could likely be improved, given normative data on eye-movement patterns. Note also that, unlike some previous eye-tracking applications[?], we did not explicitly identify saccades. Instead, classifications were based on the raw gaze data. The additional complexity of identifying saccades was found to be unnecessary, and was a source of additional noise when using eye-trackers with low temporal resolution (< 500 Hz).

Automated classifiers were also used to ensure that participants fixated calibration stimuli. However, the calibration ('log-likelihood') classifier differed from that described here for test targets (See *S2.4. Eye-tracker calibration*).

S2.8. Screen (luminance) calibration

For a given screen location, input command levels were mapped to the output luminance of the display screen, via an empirical input-output ('gamma') function (**Figure S7A**). Empirical measurements of luminance were performed at 17 input levels[ii], using a ColorCal MK II

[ii]Although 17 measurements is sufficient for most applications, future calibrations will be made using 1024 measurements, to account for local non-linearities in LCD screens.

colorimeter (Cambridge Research Systems, Cambridge, UK), and were repeated three times. A spline function was then fitted to the 17 mean-luminance values. This function could be used to predicted the necessary command level require for a specified output luminance.

LCD screens are notoriously non-uniform in luminance, with input-output functions varying as a function of screen location[?]. The chosen screen (EIZO CG277) corrects for much of this error in firmware, based on factory-made measurements. Residual error was corrected for in software, using photometric measurements made manually within lab. Input-output functions were measured independently at 48 screen locations (**Figure S7B**). Two-dimensional tensor-product linear-interpolation was then used to compute the appropriate calibration for any/every screen location (pixel).

Calibrations were validated using the ColorCal MK II photometer, and also with a CS-100 Chroma Meter (Minolta Camera Co., Osaka, JP).

Notably, the CS-100 is a handheld spot-photometer with an optical zoom. It could therefore also be used to quantify the effects of viewing angle. Even at the most eccentric test-angles (± 30°) the drop-off in luminance output from the IPS panel was minimal. Therefore, while viewing-angle effects could be corrected for in future iterations of the test, not doing so is unlikely to have affected the present results substantively.

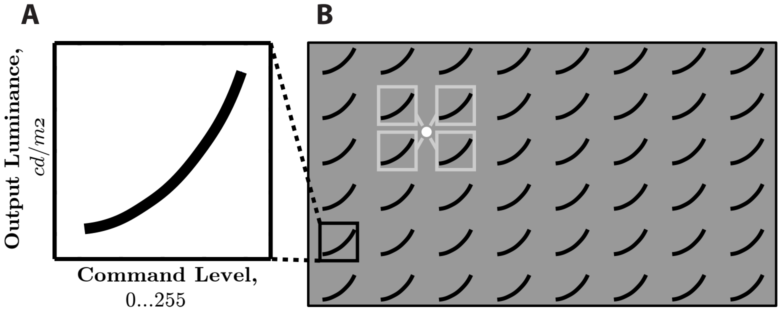

Fig S7. Screen (luminance) calibration. **(A)** To ensure uniform luminance across the screen, luminance (cd/m^2) was measured for 17 command levels (0 . . . 255), at each of 48 screen locations. **(B)** Tensor product linear interpolation was used to derive spline-fit gamma functions for every screen location (pixel).

S2.9. Using bootstrapping to perform statistical comparisons

A non-parametric boostrapping procedure was used to evaluate differences in the 95% Coefficient of Repeatability [CoR_{95}] between the present test and the HFA. In short, 64 paired-samples of test-retest differences in mean sensitivity (MS_{run2} - MS_{run2}) were randomly drawn, with replacement, from each of the two tests. The *difference* in CoR_{95} was then computed ($\text{HFA}_{CoR95}-$ Eye-track$_{CoR95}$). This procedure was repeated 20,000 times. The p-value was then computed as 2P, where P was the proportion of these 20,000 differences that had the opposite sign to the observed difference in CoR_{95}. This procedure is similar in principle to a Mann-Whitney U test, and will also give quantitatively similar results to a t-test, in situations where a t-tests parametric assumptions are met.

S3. ADDITIONAL FOLLOW-UP EXPERIMENTS

After the main test protocol was complete, three of the original thirty-two participants completed a number of additional tests, designed to validate key aspects of the reported method. These additional tests took place approximately three months after the testing reported in the Main Manuscript, and used the same basic hardware, personnel, and procedures.

S3.1. Luminance-corrected eye-tracking procedure: Correcting for a technical error in calculation of ΔL

Due to a programming error, target stimuli for the data reported in the Main Manuscript were presented 7 dB higher than their intended differential-luminance value (ΔL). Reported thresholds were corrected, post-hoc, to account for this error. However, it meant that starting priors for the adaptive thresholds algorithms were effectively 7 dB higher than intended, and therefore substantially over-estimated observers' expected sensitivity. To assess the effects of this error, three of the original participants were subsequently retested, using the correct stimulus values and/or starting prior.

No differences in accuracy or precision were observed, compared to the results reported in the Main Manuscript. In terms of accuracy, there was no significant differences between the post-hoc corrected mean-DLS thresholds originally observed (**Figure S8**; yellow bars), and thresholds re-estimated using the correct stimulus values (**Figure S8**; maroon bars). Likewise, in terms of precision, there was no significant difference in 95% Coefficient of Repeatability, CoR_{95}, for MS or pointwise sensitivity.

However, when using the appropriate target levels, ΔL, the number of trials decreased by a mean-average of 112 (40%), and test durations decreased by a mean-average of 2.4 minutes.

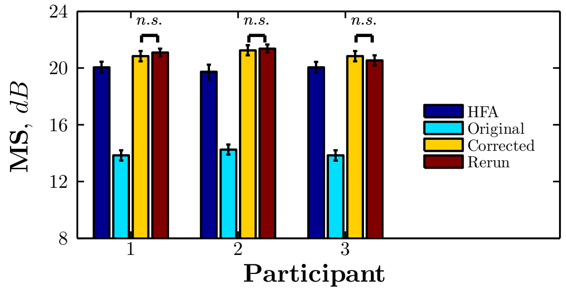

Fig S8. Mean sensitivity, MS, estimates for three individual observers, retested using intended target differential-luminance values (ΔL). Each bar represents the mean of two runs. The 'corrected' data show the original data, following post-hoc correction for a technical error. Error bars represent bootstrapped 95% confidence intervals ($N = 20,000$). Statistical comparisons represent the results of non-parametric bootstrap comparisons (see *Main Manuscript*), evaluated at $\alpha =$ 0.05.

S3.2. Substituting button-press responses for eye-movement classification

In the Main Manuscript, it was observed that false-negative response rates for Eyecatcher were significantly greater with the novel eye-tracking procedure than with the HFA. Based on informal piloting by the first author, this was mostly likely due to eye-movement classification errors, although we cannot rule out lapses in attention, or observers failing to make accurate/appropriate eye-movements.

To assess the impact of Eyecatcher's greater false-negative rate, and to clarify its cause, three of the original 64 participants were retested using a button-press as the target response. The three participants were instructed to behave as before (i.e., they continued to make eye-movements),

but were asked to also press a button when they saw a target. A 'Hit' was registered only if they pressed the button within three seconds of the target onset (irrespective of any eye-movements), otherwise a 'Miss' was registered.

Mean response-error rates for the button-press procedure and the original eye-tracking procedure are shown in **Figure S9**. False-negative errors were completely eradicated when button-pressing responses were used (0%). This is consistent with the hypothesis that the high false-negative rate was due to eye-movement classification errors. However, it was interesting to note that false-positive errors actually increased by 7% with the button-press procedure[iii]. This is perhaps unsurprising, since an observer would be much less likely to make a correct eye-movement by chance alone. It may also partly reflect button-presses being seen as a less 'costly' than button-press responses (i.e., a difference in response criterion[?]). The key corollary of this change in error rates (lower false-negative, higher false-positive) was that, in the button-press procedure, estimates of overall sensitivity, MS, increased by an average of 0.48 dB (CI_{95}: 0.43 – 0.49). Given the general pattern of response-errors, this may partly reflect the eye-tracking procedure underestimating sensitivity, and the button-press procedure overestimating sensitivity within these individuals. At the very least, however, it demonstrates that even relatively small levels of response errors can substantively affect estimated thresholds, and underlines the scope for further improvement of the test with refinement of the hardware and software.

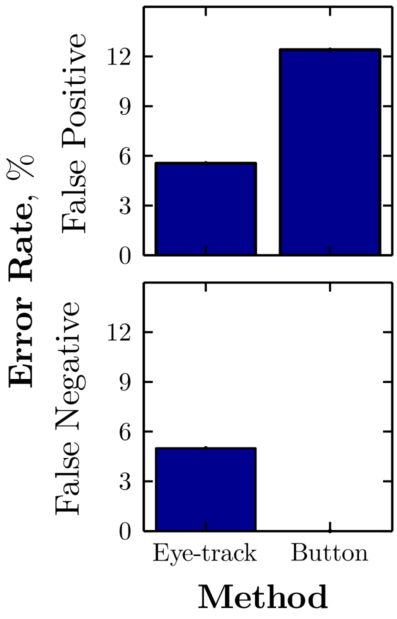

Fig S9. Mean response-error rates for three individual observers, retested using button-press responses instead of eye-movements. (Button-press false-negative error = 0%.)

[iii]NB: the observed false-positive rate under button-pressing was 12%. This is substantially greater than was observed in the main manuscript for the HFA (∼3%), which also requires an explicit button-press response. This may be due partly to the fact that the HFA does not include explicit false-positive catch trials. Instead, it estimates false-positives based on button-presses that occur during an arbitrary inter-trial 'window'. This procedure has been argued by other to substantially underestimate true false-positive rates in some observers.[?]
